# Utility of basophil activation test for predicting the outcome of wheezing in children: a pilot study

**DOI:** 10.1186/s12865-020-00395-4

**Published:** 2021-01-06

**Authors:** Jingyang Li, Jinhong Wu, Haipei Liu, Li Hua, Quanhua Liu, Dingzhu Fang, Yi Chen, Ruoxu Ji, Jianhua Zhang, Wenwei Zhong

**Affiliations:** 1 Department of Pediatrics, Xinhua hospital affiliated to Shanghai Jiao Tong University School of Medicine, 1665 Kongjiang Road, Shanghai, 200090 China; 2 Department of Pediatrics, Shanghai Children’s Medical Center affiliated to Shanghai Jiao Tong University School of Medicine, 1678 Dongfang Road, Shanghai, 200127 China

**Keywords:** Asthma, Wheezing, Basophil activation test, CD63, Children

## Abstract

**Background:**

No reliable biological marker for the diagnosis of asthma in younger children is currently available. In this study, we analyzed the differences in basophil activation test (BAT) results among children with recurrent wheezing episodes who had different asthma outcomes.

**Results:**

A prospective cohort study was conducted in children aged under 5 years who visited our pediatric respiratory clinic and ward for wheezing. After enrollment, the participants provided samples for a CD63-based BAT performed using an inhalant allergen mixture as a stimulant. Histories of personal allergic diseases and family allergic diseases were evaluated by using a questionnaire. All participants were followed up for 2 years, and their asthma outcomes were evaluated at the end of the follow-up period. The correlation between the BAT results and asthma outcomes was analyzed. Of the 45 originally enrolled children, 38 completed both the follow-up and a BAT. After stimulation with the inhalant mixture, the CD63 expression on basophils and the rate of positive CD63-based BAT results in children diagnosed with asthma were both significantly higher than those in children who were not diagnosed with asthma (*p* < 0.05 and *p* < 0.01, respectively). For the prediction of asthma, the positive predictive value and negative predictive value of CD63-based BAT was 71.8 and 69.2%, respectively. The positive likelihood ratio and negative likelihood ratio of CD63-based BAT were 1.70 and 0.3, respectively.

**Conclusions:**

Our pilot study indicates that CD63-based BAT has potential clinical value for predicting asthma outcome in young children with wheezing episodes.

## Background

Although asthma is a common chronic airway inflammatory disease in children, the natural history of asthma remains unknown. It is difficult to diagnose asthma in children aged under 5 years because of the complexity of wheezing phenotypes at that age [1]. To date, no reliable biomarkers have been developed to distinguish asthma from non-asthmatic wheezing in very young children. The asthma prediction index (API) is a practical management tool for use on young patients experiencing recurrent wheezing in clinics [2], but the sensitivity and specificity of API are limited, especially for cases in which wheezing episodes are less frequent [2, 3]. Therefore, it is urgent to explore potential effective biological markers for predicting asthma, especially in this age group.

Basophils are important immune cells that are involved in allergic diseases including asthma [4]. CD63-based basophil activation tests (BATs) are a useful tool for not only identifying specific allergens, but also monitoring immunologic homeostasis [5, 6]. BATs are used for diagnosing food and drug allergies with high specificity and sensitivity [7]. Recent research has indicated that basophil allergen threshold sensitivity tests (CD-sens), which are based on BATs, have potential use for monitoring specific immunotherapy [8, 9] and biological agent therapy [10]. Therefore, we investigated the potential clinical value of BAT results for predicting asthma in younger children in this pilot study.

The present prospective cohort study was conducted to explore the relationship between BAT results and final asthma outcomes in children with a recurrent wheeze. The clinical value of this approach for the early diagnosis of asthma in this age group is discussed.

## Results

### Subject baseline characteristics

**Fig. 1:**
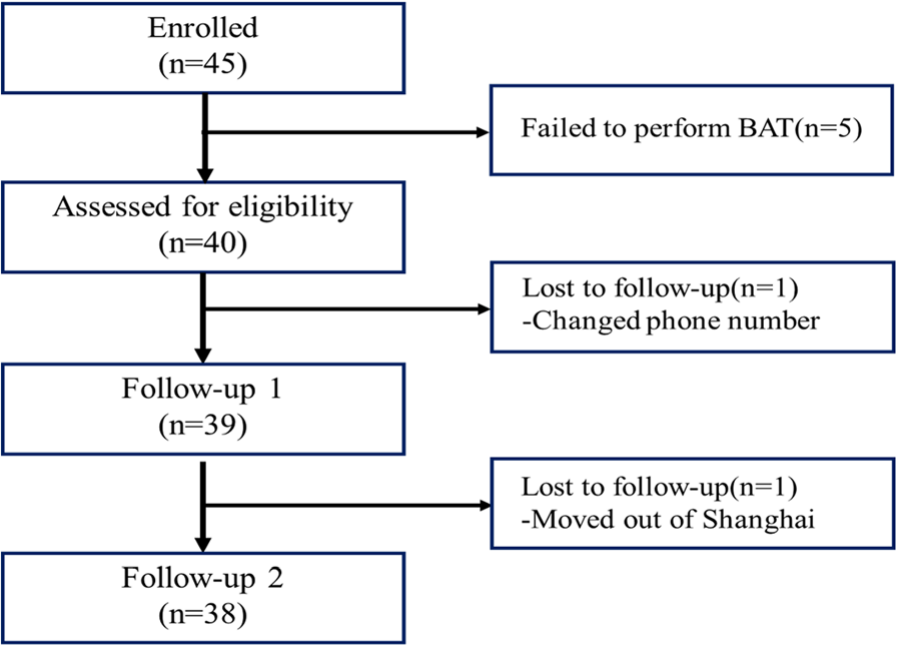
Flow diagram illustrating the study design and procedures. BAT, basophil activation test

This study enrolled 45 subjects; two patients were lost to follow-up (one changed their phone number; one moved out of Shanghai), and five patients failed to have a BAT. Thus, 43 patients completed the follow-up visit, and 38 blood samples (84.4%) were tested in BATs (Fig. 1). Of the study participants, 26.3% were female; 19 cases (50%) were aged under 3 years, and 19 cases (50%) were aged 3–5 years. Regarding patient medical history, 73.7% of the subjects had a history of atopic dermatitis (AD) or allergic rhinitis (AR), and 68.4% of the subjects were positive for food- or inhalant-specific immunoglobulin E (sIgE). Additionally, 52.6% of the subjects had family history of allergic disease. Eleven cases (40.7%) in the asthma-diagnosed group and 9 cases (81.8%) in the asthma-not-diagnosed group had lower respiratory tract infections (LRTI) when they were enrolled. Five cases (18.5%) in the asthma-diagnosed group and 4 cases (36.4%) in the asthma-not-diagnosed group received systemic glucocorticoid treatment, but none of the enrolled patients received systemic glucocorticoid treatment within 24 h before undergoing a BAT. There was no significant difference in systemic glucocorticoid use between the children diagnosed with asthma and those not diagnosed with asthma (Table 1).

**Table 1:** Comparison of demographic, atopic features and wheezing frequency according to asthma outcome

	Non-asthma (***n*** = 17)	Asthma (***n*** = 26)	***p*** value*
Sex, n(%)			
Male	7 (63.6)	21 (73.7)	> 0.05
Female	4 (36.4)	6 (22.2)	
Age, n(%)			
≤ 3 years old	8 (72.7)	11 (40.7)	> 0.05
3–5 years old	3 (27.3)	16 (59.3)	
History of personal allergic diseases, n(%)	8 (72.7)	20 (74.7)	> 0.05
History of family allergic diseases, n(%)	4 (36.4)	16 (59.3)	> 0.05
sIgE, n(%)	5 (45.5)	21 (77.8)	> 0.05
Systemic glucocorticoid, n(%)	4 (36.4)	5 (18.5)	> 0.05
Wheezing frequency, n(%)			
<4	10 (90.9)	9 (33.3)	< 0.01
≥ 4	1 (9.1)	27 (66.7)	< 0.01

Allergic disease: AR, AD and asthma; * Pearson Chi-Square test

### Outcomes

A total of 38 patients (84.4%) completed the follow-up visit and BAT. At the end of the first year of follow-up, 16 patients (42.1%) had experienced less than four wheezing episodes, and 4 patients (10.5%) had experienced more than three wheezing episodes. At the end of the second year of follow-up, 13 patients (34.2%) had experienced less than three wheezing episodes, and 17 patients (44.7%) had experienced more than three wheezing episodes. At the end of the study, after the 2-year follow-up period, 26 cases (60.5%) were finally diagnosed with asthma, and 17 cases (39.5%) were diagnosed with bronchiolitis or viral-induced wheezing. There was no significant difference in sex or age between the children diagnosed with asthma and those diagnosed with a condition other than asthma (Table 1).

### Personal history of allergic diseases, family history of allergic diseases, and sIgE in children diagnosed with asthma or a non-asthma condition

In the children diagnosed with asthma and those not diagnosed with asthma, 36.4 and 59.3%, respectively, had family histories of AR, AD, and/or asthma; this difference was not statistically significant (*p* > 0.05). Similarly, there was no significant difference (*p* > 0.05) between these groups regarding personal histories of AD or AR (74.7 and 72.7%, respectively). Details on the personal and family medical history of the subjects are shown in Table 1. The positive rates of sIgE for inhalants or food allergens were 45.5 and 77.8% in the children diagnosed with asthma and those not diagnosed with asthma, respectively; this difference was not significantly different (*p* > 0.05; Table 1).

### Performance of BAT in predicting asthma outcomes in children with wheezing episodes

**Fig. 2:**
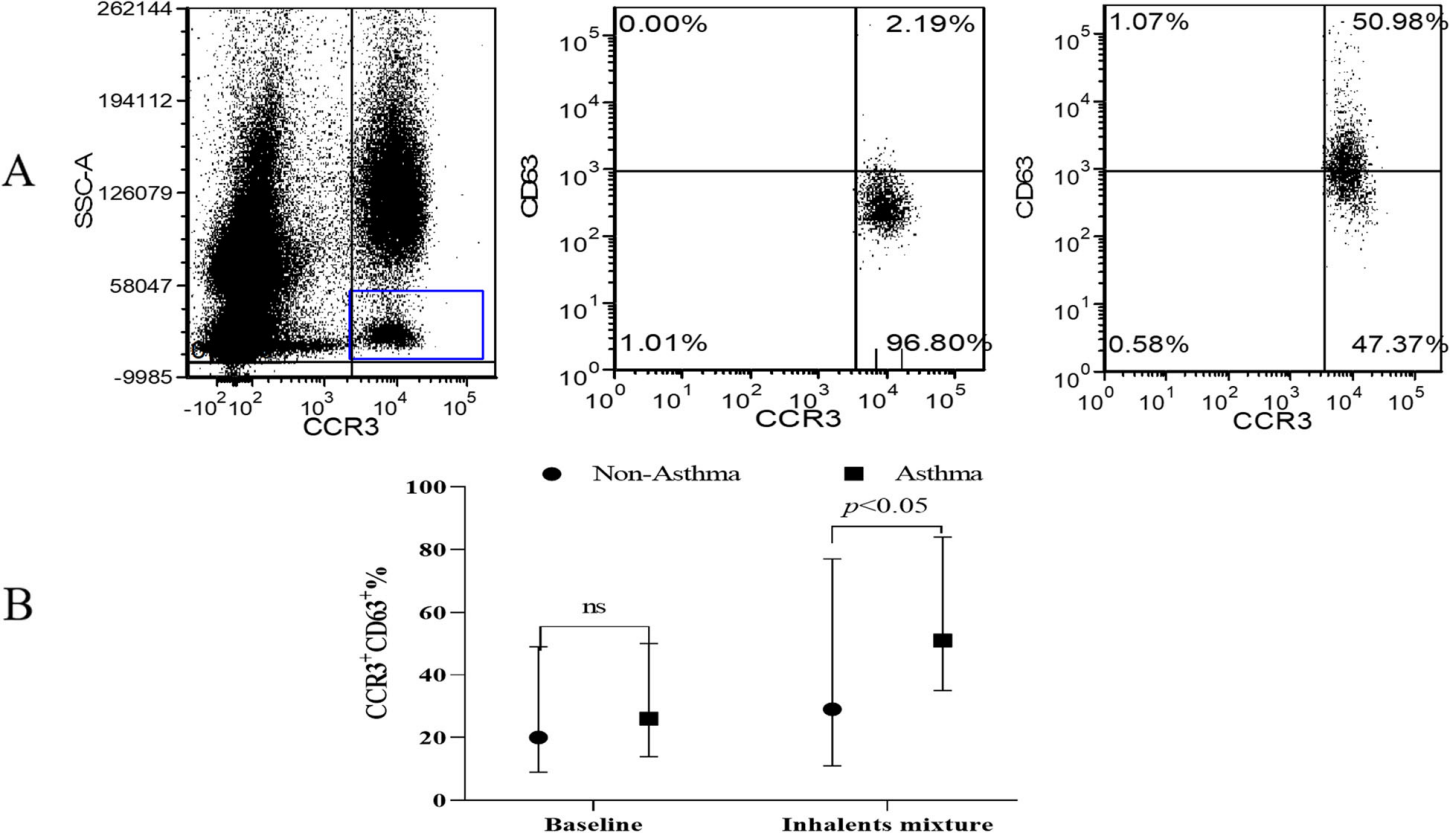
Flow cytometry anlysis of CD63-based BAT. **a** BAT was evaluated using the basophil activation marker CD63. At the time of subject enrollment, blood samples were collected, and the peripheral blood basophils were stimulated with an inhalant allergen mix. CCR3^+^ cells with low side scatter were considered basophils, and the CD63^+^ basophils were termed “activated basophils”. **b** The median levels of CD63 expression by basophils after inhalant allergen stimulation were 60.0% (34.8–84.2%) and 29.2% (11.1–77.4%) in children diagnosed with asthma and those not diagnosed with asthma, respectively. Data are expressed as the median (IQR); a Mann-Whitney rank sum test was used to compare the difference between groups (*, *p* < 0.05)

We evaluated the differences among BAT results on the basis of subject asthma outcome. At time of enrollment, a BAT was performed by stimulating peripheral blood basophils with an inhalant allergen mix. The BAT results were evaluated using the activation marker CD63. Basophils were identified as CCR3^+^ cells with low side scatter, and the CD63^+^ cells in this group were termed “activated basophils” (Fig. 2). Positive BAT results were defined as an upregulation of CD63^+^ cells of at least 15% over baseline after stimulation with inhalant allergens. The positive rate of CD63^+^-based BAT was significantly higher for the children diagnosed with asthma (85.2%) compared with that for those not diagnosed with asthma (18.2%) (*p <* 0.001, Table 2). The level of CD63 expression on basophils after inhalant stimulation was also significantly higher in children diagnosed with asthma (60.0% [34.8–84.2%]) than that in those not diagnosed with asthma (29.2% [11.1–77.4%]) (*p* < 0.05, Fig. 2).

**Table 2:** Comparison of CD63-based BAT in patients diagnosed with or without asthma

CD63-based BAT	Case, n(%)	Asthma, n(%)	Non-asthma, n(%)	***X***^***2***^	***p value***
Positive	25 (65.8)	23 (85.2)	2 (18.2)	12.755	< 0.001^*^
Negative	13 (34.2)	4 (14.8)	9 (81.8)		

* Continuity correction ***X***^***2***^

The sensitivity, specificity, positive predictive value, and negative predictive value of the API in predicting asthma outcome for our subjects were 88.9, 45.5, 80.0, and 62.5%, respectively. The same parameters for the CD63-based BAT were 85.2, 50.0, 71.8, and 69.2%, respectively. The positive likelihood ratios of the API and CD63-based BAT were 1.63 and 1.7, respectively; the negative likelihood ratios of these tests were 0.24 and 0.3, respectively (Table 3).

**Table 3:** Performance of the API and BAT in predicting the outcome of children with wheezing episodes

Predictors	Sensitivity	Specificity	PPV	NPV	LR+	***LR-***
**API**	88.9	45.5	80.0	62.5	1.63	0.24
**CD63-based BAT**	85.2	50.0	71.8	69.2	1.70	0.30

*API* Asthma Predictive Index, *PPV* Positive predictive value, *NPV* Negative predictive value, *LR+* Positive likelihood ratio, *LR-* Negative likelihood ratio

## Discussion

API is a practical tool for predicting the risk of asthma in preschool children; however, its predictive values are limited when wheezing episodes are infrequent [11]. The discovery of a biological marker predictive of asthma development would be of great clinical value. To date, several biomarkers, such as the fractional concentration of exhaled nitric oxide (FeNO), blood eosinophils, sIgE, exhaled breath condensate, and periostin, have been evaluated with varying results, but none have been ideal [12, 13].

In this pilot study, we examined the clinical value of the BAT for predicting asthma. We recruited children aged under 5 years who presented with wheezing or a history of wheezing episodes. We performed a BAT on patients’ samples at the time of their enrollment and followed the subjects for 2 years to see whether they were diagnosed with asthma during that time. We first analyzed the expression levels of the basophil activation marker CD63 at baseline and found no significant difference between children who were later diagnosed with asthma and those who were not. Because inhalants are closely related to asthma symptoms, we then performed a BAT on basophils stimulated with a mixture of inhalant allergens. The CD63 expression levels and positive rates of the CD63-based BAT were significantly higher in the children diagnosed with asthma than in those who were not diagnosed with asthma. Despite the low wheezing frequency in our subjects, the performance of the CD63-based BAT for predicting asthma diagnosis in our study was similar to that of API.

Basophils have been reported to play important roles in allergic diseases, such as asthma, AR, and AD, in animal disease models and in vitro studies [4]. Our group previously confirmed that basophils participate in Th2 immune responses and allergic airway inflammation as an important immune cell [14, 15]. Despite ongoing debate about the importance of basophils in initiating Th2 immune responses and allergic inflammation [4, 16], recently, increasingly more research has focused on the clinical utilization of BAT. This test not only has diagnostic value for the identification of specific allergen sensitization and diagnosis of food or drug allergy with high specificity and sensitivity [7], but it also has great clinical value in measuring a potential biomarker for monitoring allergen-specific immunotherapy [8, 17] and biological therapies for allergic diseases [10, 17]. Because of the many factors that influence wheezing and the complexity of the underlying mechanism of asthma, there are no known criteria for asthma diagnosis in younger children. The present study assessed a potential biomarker for the prediction and early diagnosis of asthma in young children.

In summary, our exploratory study revealed that the BAT has potential clinical value for predicting asthma diagnosis in children with recurrent wheezing episodes. However, this study had some limitations in its sample size and follow-up time; thus, the conclusions may have some limitations. Further studies with a larger sample size and prolonged follow-up time that additionally perform BAT when the subjects are free of symptoms and medication are needed to confirm our current findings.

## Conclusions

Our pilot study indicates that a CD63-based BAT has potential clinical value for predicting asthma outcome in young children with wheezing episodes.

## Methods

### Study design and population

This study was a prospective cohort observation trial conducted from July 2017 to March 2018 at Shanghai Xinhua Hospital and Shanghai Children’s Medical Center affiliated with Shanghai Jiao Tong University School of Medicine. Children aged under 5 years with a convincing history of wheezing episodes (confirmed by the researchers or documented as “expiratory wheeze” in previous medical records) were enrolled. The exclusion criteria were other coexisting chronic respiratory illnesses, e.g., bronchopulmonary dysplasia, tracheobronchial foreign bodies, congenital heart disease, or acute or chronic infectious diseases. Patients who had received immunomodulators 3 months before or during treatment were also excluded. The Ethics Committee at Xinhua Hospital approved the study. The children’s legal guardians provided written informed consent before the research began.

The children’s histories of allergic diseases (such as AD and AR as diagnosed by a specialist) and family histories of allergic diseases were obtained through detailed questionnaires completed by their parents.

### Laboratory studies

#### Serum sIgE measurement

All enrolled subjects participated in sIgE testing. Serum samples were measured via the DX-Blot 45 Automatic Western Blotting instrument (Hangzhou Zhejiang University Dixun Biological genetic Engineering Co., Ltd. Hangzhou, Zhejiang, China) as per the manufacturer’s instructions.

#### Basophil activation test

Anticoagulated peripheral blood was collected and stored at 2–8 °C for up to 6 h. The Basophil Activation Test Set (Bühlmann Laboratories, Germany) was used for the BAT. In vitro stimulation was performed using an inhalant mix (BAG-IX1, Bühlmann Laboratories) that included common inhalation allergens (GX1, G12, T3, T4, W6, W9, M6, D1, D2, E1, and E2) as specific antigens. Cell stimulation buffer (100 μl) containing FITC-labeled anti-CD63 and PE-labeled anti-CCR3 monoclonal antibodies was added to three aliquots of 50 μl of blood. Every aliquot was combined with 50 μl of cell stimulation buffer containing 250 ng/ml inhalant mix as a test sample, 50 μl of cell stimulation buffer containing anti-FcεRI Ab as a positive control, or 50 μl of cellular stimulation buffer only as the baseline control. The samples were mixed and incubated at 37 °C in a water bath in the dark for 30 min. After the red blood cells were lysed, the cells were resuspended in phosphate-buffered saline and assessed with a CytoFLEX flow cytometer (Beckman Coulter, USA). Basophils were gated as CCR3^+^ cells with low side scatter. The CD63^+^ cells in this group were termed “activated basophils”. We acquired data for at least 500 basophils, and analyzed these data with CytExpert software (version 2.3.0.84). The test result was considered positive when the level of CD63 expression in stimulated cells was > 15% over the baseline level. All BATs were performed within 6 h after blood sampling.

### Data collection

The included children were followed up for 2 years in our clinic. Patients who required controller therapy visited the clinic monthly. Otherwise, the patients visited our clinic only when they had respiratory symptoms. All patients visited the clinic at least once a year, and an annual telephone interview was conducted with their parents through questionnaires regarding wheezing episode frequency, asthma diagnosis by a pediatric respiratory specialist, and medication for recurrent wheezing. Each asthma diagnosis was made by a pediatric respiratory specialist in accordance with guidelines for the diagnosis and management of asthma in children (2016), which were developed by the Chinese Pediatric Society, Chinese Medical Associatio n[18] and Global Strategy for Asthma Management and Prevention (2017) (Chapter 6, Asthma in children 5 years and younger). For children older than 5 years of age, the asthma diagnosis was based on typical respiratory symptoms, lung function test results, and FeNO levels. For children less than 5 years of age, the asthma diagnosis was evaluated at the end of the 2-year follow-up period on the basis of recurrent wheezing, response to bronchodilator treatment, history of allergic disease, allergen sensitization, history of asthma in first-degree relatives, and clinical improvement during 3 months of controller treatment (ICS or montelukast).

All patients were assessed by API on the basis of their parents’ responses to detailed questionnaires, medical record information obtained from annual outpatient services, serum sIgE, blood eosinophilic cell counts, and follow-up results by phone. Participants with one major or two minor risk factors were defined as API-positive [19]. Parental history of asthma, physician-diagnosed AD, and sensitization to no less than one inhalant allergen were defined as major risk factors. Minor risk factors included sensitization to food allergens, eosinophil levels in the peripheral blood of ≥4%, and wheezing unrelated to a cold. Children with no major factors and no more than one minor factor were defined as API-negative.

### Statistical analysis

Participant demographic characteristics and wheezing features were summarized with amounts and percentages. Contingency tables were developed to compare the difference in qualitative variables between children diagnosed with asthma and those not diagnosed with asthma using a Chi-squared test. Continuous variables are represented by the median (interquartile range, IQR). A Mann-Whitney rank sum test was used to compare the non-normally distributed data. All statistical analyses were performed using SPSS v.22.0 (IBM SPSS Statistics, Armonk, NY, USA). A *p*-value of < 0.05 based on a two-tailed test was considered statistically significant.

## Data Availability

All data generated or analyzed during this study are stated in this published article.

## Abbreviations

BAT: Basophil activation test
API: Asthma Predictive Index
AD: Atopic dermatitis
AR: Allergic rhinitis
sIgE: Specific IgE
FeNO: The fractional concentration of exhaled nitric oxide
LRTI: Lower respiratory tract infections
